# Pursuit of Digital Innovation in Psychiatric Data Handling Practices in Ireland: Comprehensive Case Study

**DOI:** 10.2196/64919

**Published:** 2025-06-24

**Authors:** Rana Zeeshan, John Bogue, Amna Gill, Mamoona Naveed Asghar

**Affiliations:** 1 School of Computer Science, University of Galway, University Road, New Castle, Galway, H91TK33, Ireland; 2 School of Psychology, University of Galway, Galway, Ireland; 3 Mental Health Services, Ballymun Civic Centre, Dublin North, Dublin, Ireland

**Keywords:** mental health records, Irish Mental Health Services, mental health care reforms, speech-to-text transcription, blockchain application in health care, AI application in psychiatry, Ireland, psychiatric, mental disorders, behavior disorders, mental illness, mental health professionals, psychiatrists, data management, surveys, data, psychiatric data, case study design, comprehensive case study, digital, innovation, technology use

## Abstract

**Background:**

Ireland is ranked among the most disadvantageous European countries in terms of mental health challenges. Contrary to general health services that primarily focus on diagnosis and treatment, the mental health sector in Ireland deals with highly sensitive psychiatric case notes based on patient-doctor conversations. Such data, therefore, must be collected, analyzed, and stored with an approach customized specifically for psychiatry.

**Objective:**

This study’s objective involves examining the state of data handling practices in the Irish Mental Health Services (MHS), identifying the shortcomings regarding privacy, security, and usability of psychiatric case notes, and proposing an innovative technological solution that addresses most of the surfaced challenges.

**Methods:**

The study was conducted using a comprehensive methodology. Our approach involved a thorough literature review, ethics approval, web-based surveys with mental health professionals as participants, interviews of psychiatrists, interactions with mental health organizations, analysis of inspection reports by the Ireland Mental Health Commission, and comparative evaluation of existing IT solutions. The thoroughness of our adopted research methodology instills confidence in the reliability and validity of our findings.

**Results:**

Our study revealed outdated data management, heavy reliance on paperwork resulting in serious repercussions, parallel workload, alarmingly low readability of notes, and a nonviable setup that hinders research and analytical examination. Our survey reported an average score of 4.37 of 10 (SD 1.25) given by participants in terms of technology use. Regarding privacy measures, 75% (n=12) of participants mentioned that staff members are allowed to keep their phones while accessing psychiatric case notes. Similarly, 80% (n=13) of submissions highlighted that multiple staff members can access sensitive notes and patients’ contact information. On the other hand, Mental Health Commission reports showed that their inspections are limited to evaluating physical privacy only. Regarding technological comparative analysis, we observed that conventional IT solutions are vulnerable against cyberattacks and fall short in addressing multiple challenges simultaneously. Therefore, an innovative convergence of different technologies is needed. Our research supports speech-to-text transcription for data collection, interactive artificial intelligence for data analysis, and permissioned blockchain for data storage and retrieval. Our survey participants also estimated the proposed solution to optimize their workload by an average of 35%.

**Conclusions:**

Irish MHS seem to be handling psychiatric data under polycrisis circumstances; therefore, a single-dimensional digitization of records would not be sufficient in addressing the wide range of concerns. In addition to highlighting intertwined challenges in Irish psychiatry and validating the need for innovation in data handling practices in Irish MHS, this study culminated in the proposal of an innovative technological solution that offers a significant contribution to a considerably improved, efficient, and compliant service delivery in mental health care.

## Introduction

Mental health care is a critical component of public health systems, playing a pivotal role in addressing the challenges of mental disorders and promoting overall well-being. The importance of mental health care extends beyond individual health outcomes, encompassing significant social and economic implications.

According to the third annual Mental State of the World (MSW) report, Ireland ranks among the countries with the lowest mental health score globally. Specifically, Ireland’s Mental Health Quotient places it as the second worst in Europe, with the United Kingdom ranking worst, as shown in Table 1. Furthermore, Ireland is notable for having a significant proportion of respondents classified as distressed or struggling, ranging from 30% to 36% (Table 1). These findings were based on data collected from over 400,000 respondents across 64 countries in 2022, providing a comprehensive assessment of the mental well-being of included nations [1].

**Table 1.** Relevant statistics from the third annual Mental State of the World report 2022.

Countries	Mental health quotient score	Percentage of distressed population
Belgium	72.1	18.9
France	74.1	19
Spain	70.2	22.6
Germany	65.9	24.5
Ireland	56	30.4
United Kingdom	46.2	35.7

Another study conducted by scholars from Maynooth University, National College of Ireland, and Trinity College Dublin reveals that more than 40% of Irish adults experience a mental health disorder [2]. In terms of economic burden, mental health issues in Ireland are reported to be costing the country over 8.2 billion euros annually (a currency exchange rate of 1 EUR=US $1.1201 is applicable) [3]. As highlighted in the Organisation for Economic Co-operation and Development report from 2018, the cost of mental health problems accounts for 3.2% of Ireland’s gross domestic product [4]. These financial implications underscore the urgency of addressing mental health challenges within the country and investing in robust mental health care infrastructure.

Mental health services (MHS) in Ireland are responsible for handling the sensitive and private data of patients, including their medical history, diagnoses, treatment plans, and personal information. When it comes to psychiatric data, the stakes get much higher. There have been several instances of mental health data breaches, abuses, and privacy violations within Ireland and across Europe, highlighting the need for improved security measures. With the growing reliance on digital health records, the risk of data-related threats continues to increase. In 2023 alone, European Union countries reported 309 major cybersecurity incidents in the health care sector, the highest among all critical sectors [5]. We have also listed some relevant incidents of data violations in Table 2.

**Table 2.** List of relevant data violation incidents.

Country	Incident	Year
France	Two French health care payment providers, Viamedis and Almerys, experienced data breaches affecting over 33 million people, exposing personal details and insurance information [6].	2024
Romania	A ransomware attack on Romania’s health care management system has forced 100 hospitals to take their systems offline [7].	2024
Ireland	The Health Service Executive of Ireland experienced a serious ransomware cyberattack, which led to all its IT systems nationwide to be halted. The attackers demanded a ransom for not publishing private health data [8].	2021
Finland	A hack of psychotherapy records affected thousands of patients. Many of them reported instances of receiving emails with ransom demands paired with warning of disclosing their private conversations with the therapists [9].	2020
Ireland	A doctor was reported for the abuse of his patient’s contact information by sending sexually motivated text messages [10].	2018
Ireland	Irish High Court ordered the suspension of a psychiatrist over allegations of sexual misconduct with vulnerable patients based on their mental health records [11].	2013

These incidents underscore the critical necessity of comprehending the deficiencies in data handling practices within Irish MHS and the pressing need to devise an appropriate solution that effectively addresses different concerns. Considering such challenges, it was important to evaluate different technological methods that are commonly adopted for securing psychiatric data and then compare those practices with the ones in Ireland MHS. When it comes to the state of technological advancements in psychiatry, the most common solutions are electronic health records (EHRs) and personal health records (PHRs). While EHRs and PHRs are useful solutions, they come with their own set of security risks [12,13]. Similarly, there is a wide range of technological solutions involving cloud, blockchain, and conventional databases that have been adopted by health care sector all over the world. eHealth or e-therapy is another recent form of counseling that has been arising during the last few years [14,15]. The World Psychiatry Association also promotes digital means in mental health care worldwide. Moreover, it has a dedicated working group on digitalization in mental health [16,17]. Since our research picked Ireland’s MHS as a case study, we finalized our research objectives as follows:

- System insights: To gain insight into psychiatric procedures and evaluate how sensitive data are handled within the Irish mental health system.
- Stakeholders engagement: Interact and collaborate with different stakeholders, such as psychiatrists, psychologists, and mental health organizations. Document their concerns and establish a feedback loop.
- Technical proposal: Document the research findings and propose a solution that addresses the surfaced challenges after getting it validated by the stakeholders.

Furthermore, to validate and document the existing state of data handling practices in Irish MHS, this study makes a noteworthy research contribution toward technological advancement in the mental health care sector. Several methods were adopted to confirm the consistency of surfaced shortcomings. Moreover, different stakeholders of Irish MHS were engaged in research participation, public and patient involvement contribution, and critical analysis of proposed solutions. In addition to interacting with public MHS, the scope of this study was extended to relevant third-party organizations as well. Such a multifaceted approach helped us map out an innovative and novel technological solution based on consultations with the most pertinent stakeholders of the loop. This involves the convergence of different technologies, that is, speech-to-text transcription, artificial intelligence (AI), and permissioned blockchain. These technologies were selected for specific purposes with proper justifications, following an in-depth comparative evaluation against alternatives.

The remaining sections of this paper are organized as follows. The Methods section presents the methodology with pertinent details, involving interviews, surveys, third-party interactions, and technological evaluation. The Results section includes results and findings related to interviews, surveys, and descriptive comparative evaluation of different technologies. The Discussion section discusses a proposed solution, along with its applicability and novelty compared to the existing state. It also discusses the foreseen challenges and limitations of the presented work. The Conclusions section contains concluding remarks and future scope.

## Methods

### Study Design

An extensive mixed methodology was adopted to perform a thorough case study regarding existing data handling practices in Irish MHS. This research intends to document the current practices and propose an IT solution to further secure and improve existing service delivery or address any identified shortcomings. The following steps were taken for preliminary investigations, which led to the new technical proposal for Irish MHS.

### Interviews With Psychiatrists

Detailed interviews were conducted with psychiatrists working in MHS in Ireland, that is, Galway, Dublin, Cavan, and Donegal. This helped us develop a basic understanding of the Irish psychiatry sector, its challenges, and any scope for improvement.

#### Case Notes in Psychiatry

It was brought to our knowledge that a large amount of paperwork is generated each day in the Irish MHS in the form of “case notes.” Psychiatry case notes are used by doctors and nurses to plan, implement, and evaluate treatment for mental health patients. They are also used to track progress, exchange information among other health care practitioners, and ensure that care is being delivered safely, ethically, and effectively [18]. Furthermore, these notes are highly useful in the analysis of different cases. Clinicians often study these notes to identify what went wrong at what stage. Therefore, the authenticity and quality of psychiatry notes are of particular significance.

#### Reliance on Cognitive Abilities

Another aspect brought to our notice was that the existing practice relies on the human ability of a doctor to write down the conversation with a patient without skipping any important information. Such reliance on doctors’ cognitive abilities results in a lack of uniformity regarding a patient’s case record [19].

Moreover, a large percentage of these handwritten notes have quite a low readability. One of the psychiatrists claimed that 70% of the contents of psychiatric case notes are not readable. This is quite concerning, as this low-readability problem would also hinder the potential of research and analysis of such notes.

#### Legal Aspects

One interesting application of these psychiatry notes is connected to occasional legal matters. Evaluation of a suspect’s mental vulnerability during criminal proceedings is quite common in Europe [20]. Under some circumstances, the solicitors are granted access to the psychiatry notes of a patient by the courts. The solicitors then use the documented conversation and assessments to establish their points whether in favor of proceedings or against it. This is quite a serious and sensitive domain. If we connect this application with the readability and uniformity challenges described earlier, those shortcomings could potentially manipulate or affect the outcome of a legal battle.

We came across a public example quoted by solicitor Sarah Grace in an interview to the RTE Upfront program in 2023 [21], which validated this aspect.

In her words: “In Ireland, in any sexual offence case, the therapy notes, or counseling records of the victims can be used against them to disprove what they are saying in trial.”

She also pointed out that the entire defense team of solicitors as well as the accused can read those notes: “After the violation of your body, you also go through the violation of your privacy and dignity.”

#### Technology Adoption

When it comes to the existing state of technology use in the Irish MHS, psychiatrists mentioned that so far, the technology has not actually helped them, instead, it has built a parallel system (paperwork+digital tasks) and increased the workload. As articulated by a psychiatrist:

Technology was supposed to REPLACE our workload, not ADD to it.

A bit of variation was noticed in the extent of technology use among different MHS across Ireland. For example, unlike Cavan MHS, which relied largely on paperwork, the MHS in Letterkenny adopted a tool named T-Pro for the digital dictation of nursing notes, letters, and other documents [22]. Although this tool reduces time consumption in some specific tasks, it still does not replace the overall practice.

On the other hand, the digitization of mental health records should be considered beyond workload optimization, as it offers numerous benefits like enhanced case analysis and improved readability. Thus, workload reduction should be seen as an added advantage rather than a necessary requirement.

#### Patient-Doctor Equation

Psychiatrists also mentioned that several patients are not fully confident about the privacy of these notes. Some patients get uncomfortable, watching their clinician making notes about them while they are talking. They are, therefore, reluctant to share their thoughts freely, which hinders diagnosis. We also came across a similar observation made by psychiatrists in Switzerland [23], where the patients were worried about being judged or mistreated by medical staff if their report mentions their mental ailment. For some clinicians in the Irish MHS, this often results in maintaining 2 versions of notes, that is, an official version for the institution and a private version to keep track of highly confidential information related to their patients.

#### Quest for Solution

These insights put emphasis on the need for an automated technological solution to collect such sensitive data. The process should be automated to some extent, as asking doctors to replace “writing” with “typing” will only solve the readability issue. Still, it will not solve all the other challenges discussed earlier, that is, reliance on cognitive ability, workload, uncomfortable patients, authenticity, and uniformity.

We then asked, what if we record such treatment sessions (audio)? Although that could potentially solve many problems, we were informed that recording conversations would increase the sensitivity of these data by multiple folds. This discussion then brought us to speech-to-text transcription as the most reasonable potential solution, which will be discussed later in this paper.

### Community Engagement—Web-Based Survey

#### Overview

A web-based survey was also conducted to validate the preliminary findings and explore the respective scope of improvement. The target participants for our study were mental health practitioners working at Irish MHS, that is, psychiatrists, psychologists, psychiatric nurses, and senior consultants.

The inclusion criteria were mental health practitioners working at Irish MHS, who are involved in handling psychiatric case notes in any capacity.

A participant gets excluded if he or she is not a mental health practitioner or clinician, he or she has not experienced working in the Irish MHS, and he or she was not involved in handling mental health notes or patient’s data.

#### Survey Sections

The survey comprised 9 sections, with 3 to 5 multiple-choice questions each. The questionnaire did not ask for any confidential information related to any institution. The summarized breakdown of the different sections of the survey is given below:

1. Participant’s experience of working at Irish MHS (years).
2. Admission: Queries regarding the process of data handling during a patient’s admission.
3. Treatment: Queries regarding the process of data handling during a patient’s treatment.
4. Procedures: Data handling during an exchange of information between different departments.
5. Privacy and security: Measures in place to ensure privacy and security of the patient’s data.
6. Technology: Extent of technology use and familiarity with modern digital practices.
7. Workload: Queries regarding the workload of patients on practitioners based on existing practices.
8. Assumed cases: Queries regarding participant’s opinions about different assumed situations.
9. Suggestions: Submit any useful suggestion that could aid the research objectives.

### Third-Party Interactions

#### Overview

In addition to evaluating the public and mainstream MHS in Ireland, we found it important to involve the insights from relevant third parties, such as government regulatory bodies for psychiatry and private sector. We had three reasons for this outreach: (1) to paint a bigger picture that involves diverse perspectives in the case study, so we may not miss out on something significant; (2) to check and ask around whether the shortcomings surfaced during our research have already been identified or documented by some other stakeholder or not; and (3) to go through the progress made by different players in psychiatry in case the solution we are looking for might already be in practice. And if yes, to what extent?

#### Brothers of Charity Services Galway

A local mental health organization named Brothers of Charity Services (BoCS) Galway is known among mental health clinicians for having quite advanced data management software. Upon our request for a demonstration, they were considerate enough to schedule a meeting with their IT team. Some important findings and insights are highlighted in the Results section.

#### Mental Health Commission

This study also involved interacting with Ireland’s Mental Health Commission, which is responsible for promoting, encouraging, and fostering high standards and good practices in the delivery of MHS in Ireland [24]. They conduct annual inspections of MHS in Ireland and publish their reports [25]. Therefore, the Mental Health Commission’s perspective was an obvious choice to be considered and included in this case study.

### Technological Evaluation

Progressive digitization of all conventional processes and systems results in a constant generation of data at quite a rapid rate. All these data are produced, collected, processed, stored, and shared for various purposes. Many data science technologies are being adopted for health-related data handling. However, the areas of data security and privacy are not up to the mark yet [26]. We evaluated different IT solutions for mental health care data management and found that conventional data security practices are still prone to violations of different sorts.

### Ethical Considerations

Before carrying out the survey and interviews, ethics approval was obtained from the research ethics committee of the University of Galway (2023.08.024). All participants signed a consent form and were well aware of their right to opt out at any stage. No financial compensation was offered or provided to the participants. The study does not involve confidential or sensitive data collection; instead, it entails validation of routine standard practice. It is important to note that any mental health patient data were not collected, stored, or used in the study.

## Results

We have compiled results and findings from different components of the research methodology. It was interesting to notice how one problem was connected to many more.

### Interview Findings

#### Overview

The key points from our interviews with mental health practitioners working in the Irish MHS have been summarized and listed as follows: excessive reliance on paperwork and physical privacy; in addition to record-keeping, psychiatry notes are quite useful in case analysis; psychiatry notes have occasional application in legal matters; handwritten notes have alarmingly low readability and uniformity; there is a dual system in place, that is, paperwork and digital tasks; patients are often uncomfortable with this note-taking activity; patients are reluctant in sharing information to bypass the notes; patients often ask clinicians to skip some information to avoid stigma; and clinicians often maintain 2 versions of notes, that is, official and private.

#### Nonstatutory Services Report

Although BoCS’s IT solution and dashboard were comprehensive and all-inclusive, it still lacked case note–specific data handling that we sought. However, it was quite an impressive system developed over 2 decades. This is why we picked it as a reference for our investigation regarding data handling practices in the private sector of Irish MHS. Here are some highlights:

- Around 2000 staff members use their IT solution referred to as “OLIS,” that is, online information system.
- OLIS is not merely a mental health record management system, it is an extensive enterprise resource planning system with around 18 merged modules that support largely all aspects of their services. They have developed this IT system for over 20 years.
- Regarding security, the BoCS team mentioned that they have not experienced any serious cyberattack yet; however, they reported occasional phishing attacks.
- Their system does not store or manage psychiatry notes digitally. Instead, they have general fields for comments.

It is important to understand that if we remove the psychiatry notes aspect from the equation, mental health records would get the same treatment as general health records. Psychiatry notes are what make mental health records more sensitive and novel as compared to general health records.

### Survey Findings

A total of 16 submissions by mental health professionals have been collected and analyzed. A breakdown of the participants’ profiles is provided in Table 3.

Overall, the results confirm several concerns brought to our attention during the interviews. In one section, we asked all 16 participants to rank their Mental Health Department’s processes out of 10 regarding IT use and modern digital practices. The distribution of responses was as follows: a total of 2 participants rated the processes at 7 of 10, a total of 4 participants gave a score of 5 of 10, a total of 6 participants rated them 4 of 10, and a total of 4 participants assigned a score of 3 of 10. The average score of the submissions is 4.37 of 10 (SD 1.25). This is alarming enough to establish the need for digitization. Moreover, some relevant findings regarding technology use, privacy measures, and workload at Irish MHS are shared in Tables 4-6, respectively.

**Table 3.** Survey participants’ profile.

Profile	Values, n (%)^a^
Participants’ role	
Nonconsultant hospital doctors	6 (40)
Senior psychiatrists	5 (30)
Psychiatric nurses	3 (20)
Health officers	2 (10)
Participants’ role	
Working experience <5 years	50
Working experience >5 years	35
Working experience >10 years	15

a Percentages are rounded to the nearest tenth (10%).

**Table 4.** Survey findings regarding “technology use” at Irish Mental Health Services.

Question	Majority or average response	Values, n (%) ^a^
How does a general physician usually refer a patient to your Mental Health Services?	General physician sends a letter by post.	15 (90)
What information is collected at the time of admission? Is that information stored electronically or just in a patient’s paper file or folder?	Patient’s identity, contact, and residence information are collected and stored both electronically and in paperwork.	12 (75)
When patients describe their private thoughts or mental challenges to a psychologist or psychiatrist, how is that information stored?	Notes on a paper-based chart or form.	15 (90)
To what extent, are the modern technology and digital practices being used by staff members regarding mental health records?	Staff members use technology to the extent of sending emails, faxes, and scans.	10 (65)

a Percentages are rounded to the nearest multiple of 5%.

**Table 5.** Survey findings regarding “privacy measures” at Irish Mental Health Services.

Question	Majority or average response	Values, n (%)^a^
How many staff members are involved in the process of discussing a patient’s case?	5 to 10 including nonmedical support staff.	9 (60)
How often does a patient get assigned to alternative staff members, based on their availability, or shift timing?	50% split response between calling it a “routine practice” and “no such changes are made.”	N/A^b^
Which staff members are authorized to access mental health patient files?	Any staff member can access files, if needed.	13 (80)
To what extent are the staff members aware of the privacy laws and how strict are the privacy policies?	All staff members are fully aware of GDPR^c^ and relevant privacy laws, with repeated reminders or updates.	15 (90)
Are staff members allowed to keep or use their cell phones while they access patients’ records or files?	Yes, staff members are allowed to keep cell phones.	12 (75)
Could assigned staff members access the contact information of an active patient being treated by them? That is, phone number and address.	Yes, assigned staff can access patient’s contact information.	16 (100)

a Percentages are rounded to the nearest multiple of 5%.

b N/A: not applicable.

c GDPR: General Data Protection Regulation.

**Table 6.** Survey findings regarding “workload” at Irish Mental Health Services.

Question	Majority or average response	Values, n (%)^a^
On average, how many mental health patients does a psychiatrist or psychologist attend in a full working day?	5‐10	11 (70)
On average, how many hours do psychiatrists or psychologists work in a full day (officially)?	7‐9	15 (90)
On average, how much time is allocated for or consumed by each mental health patient? That is, consultation, diagnosis, report writing, and prescription.	30 minutes to 1 hour	13 (80)
On average, how early can a patient get an appointment with a psychiatrist or psychologist?	Wide range of responses; ranging between 2 days, a week, and a month.	N/A^b^

a Percentages are rounded to the nearest multiple of 5%.

b N/A: not applicable.

We shared a summarized version of the IT solution plan with our survey participants and asked them how much reduction in workload do they estimate if this plan gets implemented? Results are shown in Table 7.

From a different perspective, if we optimize the existing system, we can expect approximately 35% more patients to be accommodated each day. In our assessment, some improvements could be made by simple changes in the procedural protocols or policies, such as restricting the contact information to administrative staff only.

**Table 7.** Expected reduction in workload (survey results).

Range of reduction in workload	Participants, n
0%	0
10%‐25%	7
25%‐50%	4
50%‐60%	2
60%‐75%	3
>75%	0

### Technological Evaluation Findings

The relevant use of technology was evaluated from 3 perspectives, that is, digitization of psychiatric case notes, the scope of AI, and data storage and retrieval.

#### Digitization of Psychiatric Case Notes

##### Overview

Numerous challenges pertaining to data handling practices in Irish MHS can be effectively addressed through the digitization of psychiatric case notes. However, certain limitations persist. These concerns encompass the human limitations associated with memory retention and the time-consuming nature of manual typing during patient sessions. In light of these usability considerations, we explored the implementation of a speech-to-text transcription approach. This approach involves facilitating psychiatrists with a speech-to-text transcription tool during mental health treatment sessions.

##### State of the Art

Several digital tools have been developed for the facilitation of note-taking process. Some well-known tools include Evernote, Microsoft OneNote, Google Keep, and Apple Notes [27]. Recent progress in this industry involves the development of several audio or video recording–based software that converts the audio or video input to text format. Some notables are T-Pro, CarePatron, VoiceBoxMD, Mentalyc, Athreon, Transcription City, Upheal, etc [22,28-33]. However, such tools rely on audio or video-recorded input, which has more serious implications of its own.

This leaves us with transcription tools that process live audio data to text form without storing the sound file. Most of the tools developed for this purpose are based on different speech-to-text application programming interfaces. Some notable speech-to-text application programming interfaces are being offered by Amazon, Assembly AI, Wav2Letter, DeepSpeech, SpeechBrain, JavaScript, IBM Watson, OpenAI, Google, Intel PyTorch, etc [34-43].

### Scope of AI

Upon the successful transcription of the entire treatment session conversation between the psychiatrist and the mental health patient, we propose leveraging AI for two primary purposes: (1) to generate a summary of the treatment session and (2) to identify potential disorders and recommend appropriate treatment options.

AI has become an increasingly active area of research over the past few years in precision mental health care [44,45]. AI is expected to significantly improve psychotherapy research in the coming years. It has a lot of potential in predictive modeling [46]. Since we are dealing with the summarization of psychiatric treatment session, sentiment analysis of patient’s statements could also be quite helpful in identifying various disorders. Sentiment analysis includes natural language processing, text analysis, and computational linguistics for identifying and extracting subjective information in source materials [47].

We propose a human-supervised, query-based summarization AI model for verifying factual consistency and identifying conflicts between source documents and a generated summary. Simply put, we would keep the psychiatrist or psychologist involved in the process to verify and direct the AI-generated content before submission.

During this research, we studied the shortcomings of unsupervised automatic speech recognition [48], which refers to the challenge of generating text transcriptions from raw speech during a conversation between a psychiatrist and a patient. Another interesting component is automatic text summarization, which is becoming quite important because of the large amount of textual content that grows exponentially on the internet and various archives [49]. Automatic text summarization approaches are either extractive, abstractive, or hybrid [50]. Moreover, these days, there is a need for summarizing text based on queries.

### Data Storage and Retrieval

In response to the risks associated with EHRs and PHRs, more advanced IT solutions have been introduced. Cloud-based storage is another solution that has gained popularity in recent years. It allows for the secure storage and sharing of data over the internet [51]. Although cloud-based data handling is quite convenient and user-friendly, it could still make the records vulnerable to unauthorized access and loss of data [52].

This brought us to the doorsteps of blockchain technology, which is a decentralized digital ledger that stores data in a secure and tamper-proof manner [53]. Each block in the chain contains a timestamp and a cryptographic hash of the previous block, making it nearly impossible to alter or delete data without being detected.

Two well-known blockchain solutions were evaluated as potential platforms for our project, that is, Hyperledger Fabric and Ethereum [54]. We first compared these blockchain solutions in terms of the nature of their technology being decentralized or centralized. Although Ethereum can be categorized as decentralized and several security analysis tools have been developed for its smart contracts, they mostly have a command line interface with low usability [55]. Moreover, we found that using Ethereum could potentially violate the privacy of mental health records; therefore, Hyperledger Fabric seems more practical for us, as it is a private permissioned blockchain and is more suitable for private data [56].

Then, we compared these technologies based on their consensus mechanisms and how fast they achieve confirmations. This again resulted in Hyperledger Fabric being more appropriate because it has fewer confirmation nodes compared to Ethereum [57]. Finally, we differentiated Ethereum and Hyperledger Fabric based on throughput. This too ended in Hyperledger Fabric’s favor, as it can process far more transactions per second compared to Ethereum [57].

### Comparative Evaluation—Findings

We have categorized the findings of technological evaluation into 3 parent aspects, that is, data collection, data analysis, and data storage or retrieval. Table 8 describes what sort of solutions were available to us for each aspect of psychiatric data handling, which ones did we choose, and what are the justifications of our choices or recommendations.

**Table 8.** Comparative evaluation of different existing technological solutions.

Data handling aspects	Available solutions	Suitable choice	Justification
Data collection	Handwritten notes, tools for typing notes (Evernote, OneNote, Google Keep, Apple Notes, etc), audio or video recording tools (VoiceBoxMD, T-Pro, Upheal, Mentalyc, Athreon, CarePatron, etc), speech-to-text transcription tools.	Speech-to-text transcription tools (OpenAI, Google API^a^, JavaScript API, AWS transcribe, Assembly AI, Intel PyTorch, etc).	Digital note-taking tools would only resolve readability and digitization concerns. Whereas speech-to-text transcription would also resolve the workload, cognitive reliance, and patient reluctance concerns.
Data analysis	Multidisciplinary team meetings, written or typed reports and charts, statistical analysis tools, artificial intelligence (AI).	AI-enabled statistical analysis program with “Human in the Loop,” trained with psychiatry-specific data for general statistics, identification of patterns, and recommendations.	With the exception of handwritten notes or charts, a convergence of all available solutions is needed. AI must be involved to reduce workload and facilitate the process of analysis, diagnosis, and treatment.
Data storage or retrieval	Patient folders with case notes in cabinets, scanned or typed files in PCs, conventional databases (SQL, etc), cloud (AWS, Google, etc), blockchain (Ethereum, Hyperledger Fabric, etc).	Permissioned blockchain with hashed storage. For example: Hyperledger Fabric.	Cabinet storage does not resolve any challenge. Conventional databases and cloud services are vulnerable against cyberattacks. Blockchain is secure, distributed, and tamper-proof, which would also address authenticity, in legal aspects.

a API: application programming interface.

## Discussion

After compiling results and insights from our mixed methods, we started framing a technological solution and kept some psychiatrists in the loop to validate our technological proposals. This section summarizes the thought process behind reaching the proposed solution and how does it offer novelty.

### Proposed Approach

**Figure 1.**
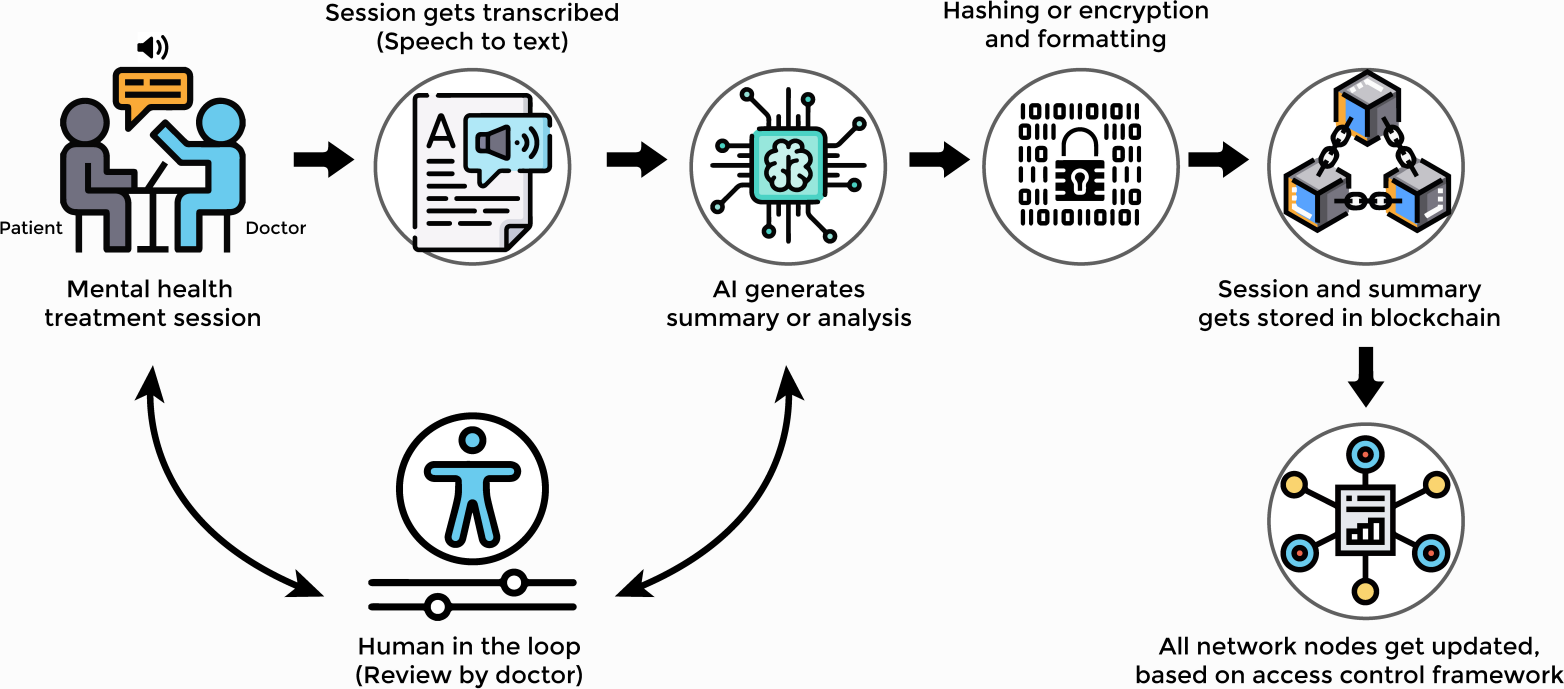
Overview of the proposed solution. AI: artificial intelligence.

The infographic overview of the proposed solution with its operations is given in Figure 1. If we further break the workflow into steps, it would be as follows:

- A mental health treatment session takes place, involving a patient and a doctor.
- During consultation, the conversation gets transcribed into digital text format.
- AI is then used to generate a summarized version or highlights of the treatment session. This step holds the potential to be advanced to probable diagnosis and treatment suggestions and insightful analysis by the AI tool.
- The AI-generated summary and analysis would then be reviewed by the doctor for any corrections or additions.
- Once approved by the doctor, the entire data would then get encrypted for security and hashed to reduce its size.
- The hashed data would then get stored to a permissioned blockchain.
- All nodes of the network would get updated based on an access control framework, that is, which staff members will have access to which sections of each patient record.

### Proposed Novelty

In Table 9, we have summarized 12 comparative aspects of the existing state of data handling practices in Irish MHS. It shows how our proposed solution would address, innovate, or improve each aspect.

**Table 9.** Comparative aspects between the current state and proposed solution.

Comparative aspects	Current state	Proposed solution
Digitization	Psychiatric case notes are largely based on paperwork in handwritten format.	Paperwork would get replaced by digitized psychiatric case notes.
Readability	Handwritten psychiatric case notes lack readability and hinder case study.	Digitized case notes would enable 100% readability and support the case study.
Cognitive reliance	Heavy reliance on the human ability to remember and write or type conversation.	Allows one to skip recalling or writing and focus on psychiatric evaluation instead.
Workload	Parallel workload (paperwork+digital tasks). If not write, one must type.	Speech-to-text transcription reduces workload and automates the note-making.
Expression reluctance	Note-writing makes patients reluctant and uncomfortable in sharing thoughts.	Automated transcription enables patients to feel comfortable while expressing.
Data storage	Patient’s data and case are written in paper files and stored in cabinets.	Data would get stored in a permissioned blockchain in a hashed format.
Data privacy	Patient’s contact data and case are explicitly described in files with stickers.	Data would be categorized in privacy terms and kept in encrypted format.
Access authorization	Unauthorized staff may access and read patient files and take photographs.	Role-based access authorization would be implemented using smart contracts.
Data tampering	Signatures are used for alteration in notes, with no digital tracking in place.	Blockchain would ensure data immutability and traceable digital footprints of all actions.
Data security	Outdated security measures for data storage and transfer, prone to cyberattacks.	Distributed yet semicentralized blockchain network would prevent cyberattacks.
Research and analysis	Handwritten notes obstruct the potential of research and psychiatric data analysis.	Digitized Psy-Notes enables psychiatric data analysis and diagnostic or treatment research.
Uniform data collection	Existing practice may result in variations in collected data by different doctors.	Transcribed treatment sessions would ensure uniformity in data for evaluation.

### Challenges and Limitations

It is important to acknowledge that the initial phase of execution of such an advanced and reform-driven technological solution is not going to be a smooth process. Each aspect of this proposed plan would involve different sorts of challenges and demands, which are as follows: lack of familiarity with emerging technologies like blockchain and AI, training needs of the medical staff regarding the adopted technological solution, need for advanced usability and human-computer interaction standards, setting up the environment and distributed network of nodes, securing investment for a required equipment and routine maintenance, employment of skillful IT professionals for development and cybersecurity, employment of a dedicated research team for tracking progress and sharing insights, reliance on speech-to-text transcription tool for accommodating different Irish accents, the accuracy of the content generated by AI, integration of the proposed solution with existing systems, and amendments to the existing policies, where necessary, such as access control protocol.

The limitations related to AI and speech-to-text transcription were scaled down by adopting a human-in-the-loop approach. However, these technologies must be improved and upgraded over time to the extent where human involvement would be reduced to formality or validation. When it comes to blockchain applications, most people are concerned about the energy consumption associated with it. It is important to clarify that the energy-related drawbacks are related to those blockchain platforms that are based on a proof-of-work consensus mechanism. Our technological evaluation has recommended a permissioned blockchain platform, such as Hyperledger Fabric, which does not involve a proof-of-work mechanism.

### Conclusions

Ireland’s mental health challenges place it among the worst in Europe. This encouraged us to conduct a thorough case study regarding data handling practices in Irish MHS, in order to contribute toward an improved service delivery in the Irish psychiatry sector. We adopted a mixed methodology to collect data from various sources for comparative analysis and cross-validation. Our study involved literature review, interviews, web-based surveys, interaction with different organizations, analysis of some relevant inspection reports, comparative technological evaluation, and a feedback loop with stakeholders.

It was noteworthy that all results were complimenting and supporting each other. However, the list of surfaced shortcomings in Irish MHS from different aspects turned up way longer than our expectations. The main concerns highlighted by results included lack of digitization, reliance on paperwork and doctors’ cognitive abilities for writing case notes, low readability, reluctance of patients in sharing their thoughts due to confidentiality concerns, sensitive legal applications, data violations by the staff, and parallel workload.

Upon brainstorming and consultation, we finalized a solution that holds the potential to resolve multiple intertwined challenges collectively. It involves collecting psychiatric data using speech-to-text transcription, then performing analysis on it using AI, which upon doctor’s review gets stored in a distributed permissioned blockchain in categorized and hashed format for retrieval based on access control rules. We shared our results and the respective proposed solution with psychiatrists working at Irish MHS, who estimated that it could reduce an average of 35% of their existing workload.

Although this proposed solution has some technical and practical challenges regarding development and usability, it is still innovative and worthy enough to be considered and executed, as it offers many positive implications. This is a novel convergence of different existing technologies that could get used smartly to address a wide range of flaws in the existing state of data handling in Irish MHS. Upon implementation, such a solution would not only facilitate the mental health practitioners in their diagnosis and treatment process but also optimize the workload and cost expenditure in the long term.

## Abbreviations

AI: artificial intelligence
BoCS: Brothers of Charity Services
EHR: electronic health record
MHS: mental health services
PHR: personal health record
